# Awareness on E‐Vape amidst increased trend and sales

**DOI:** 10.1002/hsr2.1002

**Published:** 2022-12-19

**Authors:** Amrita Ochani, Sidhant Ochani, Khushi Ochani, Simran Ochani

**Affiliations:** ^1^ Department of Medicine Jinnah Sindh Medical University Karachi Pakistan; ^2^ Department of Medicine Khairpur Medical College Khairpur Mir's Pakistan; ^3^ Department of Dentistry Dow University of Health Sciences Karachi Pakistan; ^4^ Department of Medicine Shaheed Mohtarma Benazir Bhutto Medical University Larkana Pakistan

Respected Editor,

Electronic nicotine delivery system (ENDS) a broad term which encompasses vape pens, pods, mods, e‐cigarettes and tanks are small, rechargeable, battery‐powered devices advertised as a smoking cessation aid as healthier, cheaper, and more socially acceptable. These devices produce aerosol by heating a liquid chemical or e‐juice that contains marijuana or other drugs mostly nicotine, which is addictive and is the most common active ingredient used, additionally with other chemicals such as diacetyl, volatile organic compounds, heavy metals (nickel, tin, and lead) that destroys airways in the lungs and can cause severe respiratory impairment, bronchiolitis, lung cancers and many DNA mutations.^1^, ^2^ E‐cigarette sales are rising among teens, high‐schoolers and young adults because these cool attractive gadgets with fruity‐sweet smell is gaining their attention. These are available in a variety of flavorings and becoming a new trend among them. The markets are making even more handy products like disposable vapes, high‐power vape pod kits, fast usb rechargeable vapes so that people can get quick and feasible ways to vape.^3^

E‐cigarettes are being normalized, their use is exponentially increasing worldwide and the marketing approach for these instruments is to promote them as a safer and better alternative to tobacco and conventional cigarettes regardless of environmental consequences and serious health outcomes. Some studies show that switching from combustible cigarettes to electronic cigarettes is better as they are tobacco‐free and it lowers the respiratory diseases risks (including asthma, chronic pulmonary disease, wheezing).^4^ ENDS adverse effects should not be compared with the combustible cigarettes because it has different making and contain metallic device which heats the e‐juice and release metallic (lead, chromium) ions, aldehyde and carbonyl‐related toxins in its aerosolized vapors which disturbs the host cell oral cavity hemostasis, can damage the blood vessels lining and cause respiratory and periodontal diseases. Nicotine in e‐liquid causes release of adrenaline, a hormone that raises pulse, blood pressure, and breathing rate. This could play a role in raising heart attack odds, it also disrupts brain growth and can wire the brain for addiction to other drugs, like cocaine and alcohol and even affects oral hygiene and oral health and most importantly it damages the DNA.^2^, ^5^ Aerosols exposure in pregnancy or usage of e‐cigarettes by pregnant adult can cause early abortions and will cause adverse outcomes in infants growth and development.^6^ ENDS has been mostly used in the United States and currently since few years have become more popular and common in middle eastern countries, and despite not yet been approved as a smoking cessation medication its use has increased significantly.^7^ Additionally children who never smoked but starting vaping were found to have four times higher health risks than the smokers.^8^

Middle eastern countries have highly lenient tax policies. The major ingredient of e‐cigs is nicotine which is highly addictive and their sales are not restricted and is easily accessible to the public especially to the teens. Easy availability of such products to the public promotes negative health consequences by using the product and will ultimately project habits to the future generation. Strategically regulating and banning all types of smoking including smokeless tobacco products is recommended especially in public places like institutions, restaurants, malls, and so forth. Another solution is to increase taxes in this highly consumable period which can reduce usage.^9^ Additionally, more studies and investigations are required regarding health outcomes.

## AUTHOR CONTRIBUTIONS

**Amrita Ochani**: Methodology; Resources; Writing – original draft. **Sidhant Ochani**: Conceptualization; Project administration; Supervision; Writing – original draft; Writing – review and editing. **Khushi Ochani**: Writing – original draft; Writing – review and editing. **Simran Ochani**: Writing – original draft.

## CONFLICT OF INTEREST

Not Applicable.

## TRANSPARENCY STATEMENT

The lead author (Sidhant Ochani) affirms that this manuscript is an honest, accurate, and transparent account of the study being reported; that no important aspects of the study have been omitted; and that any discrepancies from the study as planned (and, if relevant, registered) have been explained.

## ETHICS STATEMENT

Not Applicable.

## Data Availability

Data sharing not applicable to this article.
